# 儿童急性淋巴细胞白血病缓解期并发利什曼病1例

**DOI:** 10.3760/cma.j.issn.0253-2727.2022.02.015

**Published:** 2022-02

**Authors:** 慧玲 陈, 鹏云 曾, 晔 柴

**Affiliations:** 兰州大学第二医院血液科，兰州 730030 Department of Hematology, Lanzhou University Second Hospital, Lanzhou 730030, China

患儿，女，3岁，因“发热十余天”于2017年3月1日首次入院。血常规：WBC 17.7×10^9^/L，中性粒细胞占0.24，淋巴细胞占0.60，RBC 3.64×10^12^/L，HGB 89 g/L，PLT 69×10^9^/L。骨髓象：原始及幼稚淋巴细胞占0.78，糖原染色（PAS）阳性。免疫分型：异常细胞占0.765，主要表达HLA-DR、CD10、CD15、CD19、CD2、CD38、cCD79和TdT。染色体核型正常。白血病相关融合基因：TCF3-PBX1阳性，余阴性。明确诊断为急性淋巴细胞白血病（ALL）（B细胞，中危）。根据CCLG-ALL 2008方案先后予以泼尼松预激、VDLD方案（长春新碱、柔红霉素、培门冬酶、地塞米松）诱导缓解、CAM方案（环磷酰胺、阿糖胞苷、6-巯基嘌呤）早期强化、大剂量甲氨蝶呤巩固、VDLD＋CAM方案延迟强化Ⅰ、维持治疗及VDLD方案延迟强化Ⅱ治疗。化疗期间规律行腰椎穿刺及三联鞘内注射预防中枢神经系统白血病，规律复查骨髓象及微小残留病（MRD），均呈完全缓解（CR）状态。2018年1月25日，患儿因“确诊ALL 10个月余，发热6 d”再次入院，最高体温41 °C，伴有寒战、气促，偶有咳嗽，无明显咳痰，无腹痛、腹泻、胸闷等症状，家属入院前自行给予“阿奇霉素、抗病毒口服液“治疗4 d，患儿体温无明显下降。患儿无乙肝、结核等传染病史，常规预防接种，无手术外伤及食物药物过敏史，前期治疗中共输注红细胞8 U，血小板3 U。

入院体检：体温39.5 °C，脉率102次/min，呼吸23次/min，血压82/58 mmHg（1 mmHg＝0.133 kPa）。神志清楚，精神佳，无皮疹及出血点，浅表淋巴结未触及肿大。胸骨中下段无压痛。双肺呼吸音清，未闻及干湿性啰音，腹软，上腹部压痛明显，肝脏肋缘下约一横指，脾脏肋缘下约两横指，肝脾区均无压痛，移动性浊音阴性，肠鸣音3次/min。脊柱及四肢无畸形，双下肢无水肿。入院完善相关检查：WBC 2.84×10^9^/L，中性粒细胞占0.60，淋巴细胞占0.32，HGB 73 g/L，PLT 87×10^9^/L。ALT 60 U/L，AST 41 U/L，LDH 373 U/L。尿、便常规及凝血指标未见明显异常。降钙素原（PCT）9.76 µg/L。EB病毒及呼吸道病毒阴性。G试验及GM实验均阴性。骨髓象：增生活跃，未见原始及幼稚淋巴细胞。骨髓及外周血涂片未发现寄生虫。流式细胞术检测MRD阴性。胸部CT：双肺纹理增重。rK39层析实验阴性。自身抗体谱阴性。

因患儿本次入院为延迟强化Ⅱ（VDLD方案）治疗结束后10 d，目前仍为CR状态，多考虑发热由感染所致，予以对症支持治疗同时，行头孢曲松经验性抗感染治疗4 d，患儿仍有高热，抗生素升级为美罗培南。此后患儿血培养出屎肠球菌，故在美罗培南基础上加用万古霉素抗感染治疗，1周后患儿体温控制仍不理想。行多学科讨论后不排除真菌感染及耐碳青霉烯肠杆菌感染可能，抗感染方案调整为阿米卡星联合万古霉素及卡泊芬净，三药联合5 d后患儿体温仍无明显改善，而外周血三系进行性下降（WBC 0.8×10^9^/L，HGB 65 g/L，PLT 46×10^9^/L），复查骨髓及MRD仍提示ALL CR。因患儿有发热、肝脾肿大及全血细胞减少等表现，且长居地为利什曼病疫区，不排除利什曼病可能。第3次外周血涂片镜检发现杜氏利什曼原虫，且rK39抗体阳性，明确诊断利什曼病。停用所有抗生素，使用葡萄糖酸锑钠治疗3 d后患儿体温逐渐下降至正常，锑剂治疗1周后患儿肝脾逐渐回缩，血常规恢复正常，2周后肝脾回缩至正常。现患儿确诊ALL 4年余，骨髓呈持续CR状态，利什曼病无复发。

讨论：发热是血液病患者常见的临床症状，CR期患者发热的常见病因为疾病复发和粒细胞缺乏（粒缺）所致的细菌感染。对于明确诊断为感染所致发热的患者，缩短粒缺期同时行经验性抗感染治疗是有效的治疗方式。经验性抗感染失败者需根据病原学及药敏试验调整抗感染药物。本例患儿居住地为疫区，且合并发热、肝脾肿大及三系减低等临床特点，外周血涂片发现杜氏利什曼原虫，警示我们血液系统恶性肿瘤患者CR期出现发热、肝脾肿大等类似疾病复发等临床表现时，除监测原发病状态以外，还需结合患者年龄、旅居地等考虑到利什曼病等其他少见感染性疾病。

